# Gender Differences in Adolescents’ Body Complaints in Eight Countries: What Do Culture and Parents Have to Do with It?

**DOI:** 10.3390/children11101200

**Published:** 2024-09-30

**Authors:** Katharina Weitkamp, Inge Seiffge-Krenke

**Affiliations:** 1 Department of Psychology, University of Zurich, 8006 Zurich, Switzerland; katharina.weitkamp@psychologie.uzh.ch; 2 Institute of Psychology, University of Mainz, 55122 Mainz, Germany

**Keywords:** body complaints, gender differences, parental rearing dimension, family structure, adolescence

## Abstract

Objective: Although medically unexplained body complaints occur relatively frequently in adolescents, the causes are little-researched. This study examines the influence of cultural and family-related factors on somatic complaints. Methods and Measures: In a cross-cultural and cross-sectional study of 2415 adolescents from eight countries (Argentina, France, Germany, Greece, Pakistan, Peru, Poland, and Turkey), the associations of family variables with body complaints were tested and the cultural impact analyzed. Body complaints were assessed via self-reporting with a translated version of the body complaints scale of the Youth Self Report (YSR). In addition, Perceived Maternal/Paternal Behavior was assessed, as well as cultural dimensions of the eight counties. Results: Overall, females reported higher rates of body complaints than boys did. An additional negative impact of parental psychological control and anxious rearing was found that generalized across cultures, with a particularly strong impact on girls. Girls in stepparent families and boys in single-parent families reported more body complaints. Finally, body complaints were associated with Hofstede’s cultural factors in both genders, like individualism vs. collectivism, power distance, uncertainty avoidance, and masculinity vs. femininity. Conclusion: These findings are particularly important for primary care providers, as they stress the relevance of taking into account family and cultural factors in body complaints which affect boys and girls differently, to provide adequate care.

## 1. Introduction 

Body complaints are frequently reported by adolescents in both clinical and community samples. Fatigue, headache, backache, stomach ache, and nervousness/agitation constitute the most frequently occurring complaints [1,2] and were found, for example, in 76% of depressed adolescents and 33% of healthy controls. Only in about 3–10% of these body complaints could a medical cause be found [3]. Body complaints may cause significant impairment of psychosocial functioning and are strongly associated with emotional disorders in girls and with disruptive behavior disorders in boys [1,3]. Parents and adolescents are likewise disappointed by healthcare providers that did not find an explanation for their somatic complaints [4]. There is evidence in many countries that adolescents in non-clinical samples experience relatively high rates of body complaints, for example, in the nationally representative Hamburg Health Survey [2] in Germany, the Great Smoky Mountains Study in the U.S. [1], and large studies of school-based populations in Israel [5] and Pakistan [6]. In these studies, the prevalence of body complaints ranged between 31% and 64% during the last three months, with higher rates in girls than boys. Overall, females had higher levels of reported body complaints than boys. Hence, body complaints can be found in normative samples of adolescents in many countries. The question of the causes that can explain the high level of body complaints is an important one, as adolescents will seek help in the health system. However, less is known of the factors that are associated with this hidden expression of distress via body complaints. 

The developmental period of adolescence is complicated by multiple sources of life stress, including school and peer-related stress, family conflicts and dysfunction, future uncertainty, and strict communal norms [7,8]. When looking at healthy adolescent development and the emergence of body complaints in normative samples, different influencing factors need to be considered. These may be ordered through the lens of Bronfenbrenner’s ecological systems model [9], which consists of four environmental levels—the micro-system, the mesosystem, the exosystem, and the macrosystem. Each level affects the development of a young person differently. 

### 1.1. Micro Level—Family Influences

Perhaps the most important actors on the micro level of Bronfenbrenner’s model are the parents, who play a significant role in the well-being of their adolescent children. Research has consistently found parenting dimensions such as warmth, nurturing, acceptance, and responsiveness as important factors in fostering well-being throughout adolescent development, a finding corroborated in several international comparisons [10]. More specifically, a lack of parental support and high rates of parental psychological control are associated with the expression of depression and anxiety [11,12]. These symptoms are linked with body complaints in the dimension of internalizing disorders. Intrusive parenting is thought to be especially harmful during adolescence, as it often interferes with the adolescent’s development of autonomy [13]. Exposure to emotionally manipulative and intrusive parenting is likely to decrease adolescents’ confidence in expressing their own views and may instead lead to internalizing and somatic symptoms [14]. Relatedly, a parenting style was described in Europe and the U.S. called helicopter parenting, which is high on support, high on control, and low on autonomy-giving [15,16]. Driven by parental separation anxiety and aimed at limiting adolescents’ increasing separation and independence, helicopter parenting also has a negative impact on adolescent health [17]. The adverse role of non-supportive, controlling, and intrusive parenting has mostly been studied in Western samples, and little is known about the extent to which the negative impact of parenting practices may contribute to the high rate of body complaints seen in non-clinical adolescents around the globe [12]. 

An additional micro-level factor is the family status (i.e., living in a two-parent versus single-parent family or stepfamily), which may affect boys and girls similarly. Overall, the well-being of adolescents in families of divorced parents or stepparents is significantly impaired [18]. So far, previous studies focused on internalizing and externalizing symptoms, whereas the impact on body complaints has not been investigated to date. Further, the meta-analysis by Buist and colleagues [19] demonstrates links between sibling relationships and internalizing (including body complaints) and externalizing symptoms. Thus, growing up with siblings could be important to explain high rates in body complaints. Western research emphasizes more stress in girls from families with too many siblings. A connection with body complaints may exist too, and it is unclear whether this generalizes across countries [20].

### 1.2. Exosystem—Parent Workplace or Community-Based Resources

Over and above parental rearing dimensions, parents’ work status may have an impact, which is framed as the exosystem in Bronfenbrenner’s model. Parental employment is a system where the child does not belong but that influences their micro-systems, like home. Thus, parent workplace could be a factor with an indirect influence on adolescent stress and body complaints. In the National Longitudinal Study of Adolescent Health, the mother’s working status was more influential on negative child outcomes for younger children than the father’s working status [21]. This effect diminished during adolescence in single-parent families, potentially because of increases in family income due to maternal employment [22]. 

### 1.3. Macro Level—Cultural Influences

On the macro level, cultural variations may influence the reporting of body complaints. Unexplained body symptoms might serve as an indirect mode of expressing distress. Past research shows a higher likelihood of reporting distress through body symptoms compared to verbal expression among members of non-Western cultures [23]. For instance, due to severe social stigma attached to mental health problems, the somatic expression of psychological distress is more acceptable in China than the expression of anxiety or depression and elicits greater social support [24]. The concept of ”Western psychologization” has been suggested to counterpart “Asian somatization” [25]. However, the explanations for cultural differences in body complaints are more complex, considering the distinction in linguistic features of the language, the stigma associated with psychiatric conditions, or the differences in emotional expression norms across cultures.

For a more fine-graded approach, our evaluation of the association between culture and body complaints is based on Hofstede’s model of cultural dimensions [26]. The model distinguishes six dimensions along which cultural values can be compared between different cultures: individualism vs. collectivism, power distance (strength of social hierarchy), indulgence vs. restraint, masculinity vs. femininity (task orientation versus person orientation), uncertainty avoidance, and long-term orientation vs. short-term orientation. To date, Hofstede collected country-level data for over 120 countries, and each country can be positioned relative to other countries by a score on each dimension. The dimensions are statistically distinct and do occur in all kinds of combinations, although some combinations are more frequent than others [27].

In exploring the association between body complaints in adolescents and the macro-level influence of culture, several cultural dimensions seem to be particularly relevant, such as individualism vs. collectivism, power distance, uncertainty avoidance, and masculinity vs. femininity [26,27]. Where European/Western cultures place more emphasis on individualistic goals and value independence, more collectivistic cultures, like Middle Eastern and South American cultures, place more emphasis on interdependence. Individual behavior in collectivistic cultures is guided more by mutual obligations, following traditional authority figures, and maintaining harmonious relationships with close others [28]. Higher values of collectivism and power might be perceived as an obstacle to the adolescent penchant for independence. Further, cultures vary in the degree that less powerful members of society accept the unequal distribution of power [27]. Similarly, cultures high on uncertainty avoidance have rules that are more formal and less tolerant for deviant persons and groups [27]. Finally, masculine societies encourage assertiveness, competition, and material success. In contrast, feminine societies place greater emphasis on cooperation and quality of life considerations and de-emphasize competition and personal success, which might discourage separation and individual growth [29]. In sum, an inspection of the meaning of Hofstede’s cultural dimensions for adolescent autonomy development suggests that their association with adolescent well-being is worthy of closer examination. We suspect that the expression of physical symptoms is influenced by these cultural dimensions, and that the cultural perspective provides additional information on the individual expression of body complaints and should therefore be taken into account.

### 1.4. Current Study and Hypotheses

This survey study focuses on the ways in which family variables (such as parental rearing dimensions, family structure, and parental employment status) and cultural factors are associated with the expression of body complaints among adolescents from eight countries. Accumulating studies on adolescent development and health have shown the importance of parental rearing practices in adolescent psychological well-being. However, less is known of the extent to which different parental behaviors and other family-related variables are related to body complaints. Similarly, enormous variations in externalizing and internalizing symptoms have been found in adolescents around the globe [30,31], but much less is known about the cultural impact on body complaints. Finally, it is unclear what role cultural factors play beyond perceived parental behavior. 

This study examined somatic complaints of adolescents living in different cultural contexts around the world. We included three developing countries (Pakistan, Argentina, and Peru), which can be considered “tight cultures”, e.g., characterized by strong parental norms compared to “loose” Western cultures, but may also vary in strictness [32]. Since Peru and Argentina presumably share similar family values, it was important to us to investigate cultural influence in geographically close countries. Further, we selected two countries with recent political and economic changes (Turkey and Greece), which are characterized by relatively strong parent–child bonding. We further included three countries from Europe (France, Germany, Poland), where parenting practices may potentially vary despite their geographical closeness. On a descriptive level, the eight countries showed specific patterns when looking at Hofstede’s cultural dimensions [26]. The power distance index was notably higher in France, Peru, Poland, and Turkey, while Germany exhibited the lowest scores. Individualism was found to be highest in France, Germany, and Poland. The variation in masculinity across the eight countries was quite small, with slightly higher scores observed in Germany and Poland. In contrast, uncertainty avoidance scores showed more variability, with the highest values in Greece. 

We restricted our samples to sizes of about 300 per country, to the age of 15 years, and to pupils attending classes. Thus, the adolescents we investigated lived in comparably modern urban contexts, but there may be differences in parenting style, which needs to be considered if we want to look at culture-specific differences and their impact on somatic complaints. 

To examine the relationships of parenting, cultural influences, and youth somatic complaints, we first characterized the eight cultural samples (Argentina, France, Germany, Greece, Pakistan, Peru, Poland, and Turkey) according to differences in four Hofstede dimensions which seemed most relevant, e.g., we evaluated whether they differ in their position with regard to power distance, individualism vs. collectivism, masculinity vs. femininity, and uncertainty avoidance. Secondly, we explored differences in body complaints and in father’s and mothers’ parental rearing dimensions (support, psychological control, and anxious rearing) across the eight cultural samples. 

Our analyses are exploratory given the paucity of direct research on the relationship between body complaints and parental rearing dimensions across different cultures. In terms of Hofstede’s dimensions, we expected that in countries high in power distance, high in uncertainty avoidance, and high in masculinity, higher levels of body complaints would be reported. As there is research suggesting strong gender differences, with higher levels of body complaints in girls compared with boys in some countries, we intend to analyze the predictors in separate models for boys and girls. In terms of parental rearing dimensions, we expected that psychological control and anxious rearing, which have been found to be risk factors for psychological health in adolescents from Western countries, would also have a negative effect on physical health, e.g., would be associated with higher body complaints in adolescents from non-Western countries.

In the current study, we focus on the role of parenting behavior on adolescent adaptation. Yet, above and beyond the parental rearing dimension, the family status (living in a two-parent or single-parent family, living in a stepfamily, number of children, and parents’ employment status) has been found to be associated with adolescent well-being [33] and even carry over into adulthood [34]. However, less is known about the extent to which such family variables might be related to adolescent somatic complains. For this reason, family dimensions such as family status and family economic strain were added as control variables in the current analyses. 

## 2. Materials and Methods

### 2.1. Sample 

The sample consisted of 2415 adolescents living in eight countries (Asia: Pakistan; Europe: France, Germany, Greece, Poland, and Turkey; South America: Argentina, Peru). See Table 1 for demographics for each country separately. Mean age was *M* = 15.33 (*SD* = 0.61) with 56% females. The samples from the different countries varied regarding family structure and size. Two-parent families were more prevalent in adolescents’ families in Turkey (92.2%), Pakistan (87.8%), and Greece (87.3%), compared with, for instance, adolescents from France (66.5%). Additionally, the number of children per family exhibited significant variability among samples, with the lowest averages recorded in Germany (*M* = 1.11; *SD* = 0.98), Poland (*M* = 1.35; *SD* = 1.17), and Turkey (*M* = 1.23; *SD* = 1.01), while the highest average was observed in Pakistan (*M* = 3.39; *SD* = 1.71). Furthermore, perceptions of standard of living differed markedly among the samples from the different countries. Standard of living (adolescent self-report) was notably low in Argentina (2.5%) and Peru (11.1%). Forty-two percent of adolescents from Poland, on the other hand, reported their standard of living to be high when comparing it to others, followed by about a third in Turkey, Greece, and Pakistan. In Germany, 24.8% considered themselves to have a low standard of living. 

### 2.2. Procedure

Within the cross-sectional survey, we collected data from school-attending adolescents in eight different countries. The data set led to further publications in the context of adolescents’ health [35,36]. 

To ensure cross-cultural validity and equivalence, we held regular meetings with the collaborating researchers from the eight countries. As part of these consultations, the questionnaire items were translated into the official language of each country and subsequently translated back into English. Any discrepancies between the different language versions were reconciled in a stepwise process. Data collection took place in high schools of university cities to minimize variance attributable to differences in educational levels and urbanization. At the time of data collection, the ethics committee did not yet have any requirements that studies had to submit an ethics application. Obtaining informed consent from the parents was sufficient. The study was conducted in accordance with the Helsinki Declaration. Additionally, authorization to conduct the study was obtained from school district supervisors in each country. Consent forms were distributed to the participants’ parents before the assessments. We received written parental consent for 96% of the adolescents. A further 5% of potential participants were absent on the day of data collection, leading to an overall participation rate of approximately 91%. Data collection took place together within the classroom, with each adolescent receiving an anonymized questionnaire package during a designated classroom period. 

### 2.3. Measures

*Body complaints.* We assessed the participants’ level of body complaints with the respective scale from the Youth Self Report (YSR) [37]. The YSR is a self-administered scale to measure problem behavior, utilizing a response format from 0 = not true, 1 = somewhat or sometimes true, to 2 = often or very often true. The YSR covers broad syndromes of externalizing (e.g., delinquent, aggressive) and internalizing problem behaviors (e.g., body complaints, anxious/depressed). For the present study, we focused on the body complaints scale, covering the following eight items: stomach aches, tiredness, feeling tense, feeling physically weak, headaches, rashes/skin problems, nausea, and dizziness. The YSR is a widely used instrument with very good reliability [30]. In the current study, Cronbach’s alpha was α = 0.90 across countries.

Perceived Maternal/Paternal Behavior was assessed with three distinct scales, each covering a relevant facet of parental behaviors as viewed by the adolescent. Each scale was filled in separately for paternal and maternal behavior. Each item is rated on a five-point Likert scale ranging from 1 = strongly disagree to 5 = strongly agree. To measure parental support, we used five items from the Adolescent Family Process measure (AFP) [38]. To measure psychological control, we used six items from the Psychological Control Scale—Youth Self-Report (PCS-YSR) by Barber [39]. Additionally, to assess dimensions associated with helicopter parenting—also referred to as hovering—we employed the instrument developed by Kins et al. [11], which captures anxious rearing with six items. Reliability coefficients for the current sample were Cronbach’s α = 0.83 for parental support, α = 0.79 for parental psychological control, and α = 0.81 for anxious rearing.

*Cultural dimensions* were taken from Hofstede’s six cultural dimensions [26]. We used four dimensions that seemed particularly relevant for adolescent development: *individualism* vs. *collectivism*, *power distance*, *masculinity* vs. *femininity*, and *uncertainty avoidance*. The scales range from 1 to 100 (with some empirical exceptions). These dimensions distinguish preferences of different cultures on the country level. Data collected by Hofstede and colleagues spans over 110 countries and is publicly available for scientific research purposes [40]. 

Furthermore, we collected sociodemographic data (gender was assessed in a binary fashion), such as parental education, parental employment status (full-time, part-time, unemployed, in education, retired, housekeeper), self-rated standard of living (three-point scale: compared to others high, average, low), age, gender, number of siblings, and family structure (two-parent, single-parent, stepparent, foster parent family). 

### 2.4. Data Analyses 

We analyzed differences in body complaints among countries using an analysis of variance (General Linear Model) for boys and girls separately, with country as the independent variable and body complaints as the dependent variable. Additionally, we checked overall differences between boys and girls. In order to analyze the association between adolescent body complaints and parental rearing practices, we carried out hierarchical regressions with somatic complaints (YSR) as the dependent variable. We entered predictors in blocks, beginning with control variables. Thereafter, we entered predictors in-line with Bronfenbrenner’s ecological systems model, beginning with immediate micro-level influences to remote macro-level cultural influences [9]. In block 1, we entered gender, age, standard of living, parental level of education, and family status, followed by maternal rearing dimensions in block 2. In block 3, we entered paternal rearing dimensions. In block 4, we entered Hofstede’s cultural dimensions. We checked the data for multicollinearity and inspected residual plots visually to assess data fit. We calculated inter-correlations of predictors and dependent variables. Prior to the analyses, we centered the predictors (i.e., parenting variables) and created dummy variables for nominal variables (country, gender). We set the level of significance to the conventional α = 0.05 (two-tailed) and used SPSS 22.0 for data analyses.

## 3. Results

### 3.1. Country Differences in Body Complaints by Gender

Regarding the mean level of reported body complaints among countries, we found significant differences among countries for both girls (*F* = 78.345; *p* < 0.001; η^2^ = 0.294) and boys (*F* = 56.562; *p* < 0.001; η^2^ = 0.284) in the analysis of variance. Overall, young people in Greece and Turkey reported the lowest scores of body complaints, followed by Argentina, France, and Poland. In Germany, Peru, and Pakistan, young people reported the highest scores of body complaints (see Table 1).

### 3.2. Gender Differences in Body Complaints

Within an analysis of variance, we found that the level of body complaints differed significantly by gender (*F* = 89.246; *p* < 0.001; η^2^ = 0.037). In general, boys reported fewer somatic symptoms than girls. 

### 3.3. Predictors of Body Complaints in Boys 

We present the final model of the hierarchical regression analysis for boys’ somatic body complaints in detail here; for details regarding the individual steps, see Table 2. In the first block, we entered sociodemographic data as control variables: age, intactness of families, parental employment status, and perceived standard of living. These control variables alone explained a significant amount of variance (R^2^ = 0.03). Significant predictors in this initial step were two-parent vs. single-parent status and mother’s employment status. Throughout all steps, single-parent status retained a small, positive significant effect, indicating that compared to two-parent families, boys in single-parent families reported more body complaints. Additionally, a mother’s employment status was related to higher levels of somatic symptoms compared to a mother’s unemployment. In the second step, we entered adolescent-rated maternal rearing dimensions as predictors; it explained a small yet significant additional amount of variance in somatic complaint scores (R^2^ change = 0.04), although it was maternal psychological control that seemed primarily relevant in predicting body complaints in boys. In the third step, we entered paternal rearing dimensions. These predictors explained another small but significant amount of additional variance (R^2^ change = 0.04), again with psychological control serving as the relevant predictor, as well as paternal support. Both maternal and paternal psychological control were significant positive predictors, indicating that higher levels of perceived psychological control from fathers and mothers were associated with higher levels of body complaints. Additionally, paternal support was negatively associated with body complaints, meaning that boys who experience more paternal support reported lower levels of body complaints. In the final step, we entered Hofstede’s cultural dimensions into the regression analysis; this addition led to a significant increase in the amount of variance explained (R^2^ change = 0.14), with individualism, uncertainty avoidance, and masculinity as significant predictors in this block, with moderate to large effect sizes (with the exception of power distance, which was unrelated). The dimension of individualism was also a significant negative predictor of body complaints in boys (β = −0.20). Higher levels of individualism, the cultural understanding that individuals have to look after themselves, were associated with lower levels of body complaints. Additionally, masculinity was a significant predictor (β = 0.27). In countries high in masculinity, e.g., distinct gender roles, boys reported more body complaints. Uncertainty avoidance was negatively associated with body complaints (β = −0.48). Boys in countries with higher levels of uncertainty avoidance reported lower levels of body complaints. Overall, the final model explained 24.1% of variance. 

### 3.4. Predictors of Body Complaints in Girls 

We present the final model of the hierarchical regression analysis for girls’ somatic body complaints in detail here; for details regarding the individual steps, see Table 3. In the first step, we entered sociodemographic data as control variables: age, intactness of families, parental employment status, and perceived standard of living. This explained a significant amount of variance (R^2^ = 0.06). Throughout all steps, age and stepparent status retained small significant positive effects, indicating that older girls and girls in stepparent families reported more body complaints. Additionally, a higher standard of living was related to fewer body complaints. Significant predictors in this step were age, two-parent vs. stepparent family status, and standard of living. In the second step, we entered maternal rearing dimensions as predictors; this added significantly, but to a small degree, to the amount of variance in somatic complaint scores (R^2^ change = 0.04). Maternal anxious rearing and support were primarily relevant in predicting body complaints in girls. In the third step, we entered adolescent-rated paternal rearing dimensions. These predictors explained a significant amount of additional variance (R^2^ change = 0.02), again with anxious rearing and paternal psychological control being the most relevant predictors. In terms of parenting dimensions, fathers’ and mothers’ rearing dimensions were associated differently: whereas higher levels of maternal anxious rearing were associated with higher levels of body complaints, less maternal support was associated with more body complaints. For paternal rearing, higher levels of paternal anxious rearing were negatively associated with body complaints. Additionally, paternal psychological control was positively associated with body complaints, indicating that more perceived control was related to higher levels of body complaints in girls. In the final step, we entered Hofstede’s cultural dimensions into the regression analysis; the addition of these predictors led to a significant increase in the amount of variance explained (R^2^ change = 0.10). All dimensions were significant predictors in this step/block, with small to moderate effects. In terms of Hofstede’s cultural dimensions, power distance was positively associated with body complaints, indicating that in cultures with higher acceptance of power distance, e.g., the acceptance and expectancy that power is distributed unequally, girls reported more body complaints. Individualism was also a significant negative predictor of body complaints. High levels of individualism were associated with lower levels of body complaints. Additionally, masculinity was a significant predictor. In countries high in masculinity, girls reported more body complaints. Finally, uncertainty avoidance was negatively associated with body complaints, indicating that in countries with more uncertain situations, girls reported fewer body complaints. The total amount of variance explained in the final model was 21.0%.

## 4. Discussion

In the current study on adolescents from eight countries, we explored which cultural and family factors contribute to the expression of psychological distress in the form of body complaints. Drawing on the ecological systems model [9], we identified relevant factors on the micro-system, exosystem, and macrosystem level. Regarding the cultural dimensions described by Hofstede [26], the findings suggest that the expression of body complaints in boys and girls is associated mainly with high collectivism, masculinity, and uncertainty avoidance. In addition, the detrimental effects of parental psychological control and anxious rearing generalized across cultures as well. Conceptually, it may be argued that a family atmosphere and a culture that do not support adolescent independence were more likely to be associated with higher levels of adolescent body complaints, and that these relationships are different for girls and boys [41]. 

The incorporation of Hofstede’s cultural dimensions [26] was helpful in teasing out the effects of culture on adolescent adaptation, which is in line with the macrosystem level of the ecological systems model [9]. The eight countries involved (Argentina, France, Germany, Greece, Pakistan, Peru, Poland, and Turkey) showed substantial variation among them in each of the Hofstede dimensions, pointing to the unique characteristics of each country. Countries varied in individualism, but we found less variation in power distance and masculinity. 

For boys, three cultural dimensions (e.g., individualism, uncertainty avoidance, and masculinity) substantially added to the amount of variance explained over and above family-related factors and parental rearing dimensions. For girls, all four cultural dimensions were predictive, but the total amount of variance explained was higher for boys than for girls. Most associations followed the expected directions, with the exception of uncertainty avoidance. Overall, associations were in the same direction for boys and girls. We expected a stronger association with power distance because there is research showing that the habit of suppressing emotional expressions is higher in countries with strong hierarchies and a focus on male-attributed values, like competition and assertiveness [42]. Surprisingly, this cultural dimension did not play an important role in the prediction of body complaints for boys. Further, Hofstede’s cultural dimension of power distance was positively associated with body complaints in girls. Intuitively, one would expect that the experience of, in Western terms, “unfair” power distribution would be related to more psychological distress and thus more body complaints in both genders. Apparently, this effect was only found in girls who seemed to be more sensitive to an uneven distribution of power. 

In cultures high in individualism, fewer body complaints were reported from adolescents of both genders. In countries high in masculinity, boys and girls reported more body complaints. Finally, uncertainty avoidance was negatively associated with body complaints, indicating that in countries with more uncertain situations, girls and boys reported fewer body complaints. It seems cultural climates with more formal rules, a sense of knowing “the truth” [27], and less tolerance for non-normative individuals and groups seem to have either a protective factor on body complaints or keep young people from expressing these complaints within a questionnaire study. Taken together, the findings of our study demonstrate the strong association between cultural factors and the international variation in body complaints, and that the prevailing cultural scripts seem even more influential for girls.

Similar to other studies [2,43], we found a predictive value of sociodemographic variables, more specifically parental marital and employment status, which vary in its association by gender. Across all cultures, for boys, coming from a single-parent family and having a working mother was associated with higher reports of body complaints. Potentially, in a mother-headed family, especially when the mother is employed, a son may be more hesitant to openly express discomfort, loneliness, or conflicts. As a result, he may resort to communicating his distress through body complaints, which may serve the purpose of seeking support and care from his working mother. The finding that body complaints frequently result in more support from the family has been substantiated in several studies (as an example, [24]). 

The association of these factors is somewhat different in girls. Consistent with other studies from diverse cultural backgrounds, girls reported higher rates of body complaints than boys [1,2,6]. In addition, living in a stepfamily was associated with higher body complaints in girls. It may be that daughters feel uncomfortable in expressing their thoughts openly within stepfamilies. Instead, they may resort to non-verbal forms of communication, essentially “speaking with their bodies” by expressing body complaints. It is conceivable that females’ stronger emotional regulation in step-father families may play a role, as Sun and Lau [42] propose, in that the suppression of negative effects involves a continuous focusing of attention toward the self to prevent the activation of open expression. Taken together, it may be argued that in certain familial (and cultural) conditions, particularly in a position of dependency, the expression of distress in the form of body complaints instead of open communication is an understandable reaction.

Another important finding of the current study is the association between certain parenting dimensions and body complaints across cultures. Earlier studies in Western countries reported that intrusive parenting often interfered with the adolescent’s development of autonomy, leading to a greater dependency on parents, social isolation, and depressive symptoms [13]. Our study extends these findings and shows that dysfunctional parenting predicted body complaints in both boys and girls alike. Our findings seem to suggest that boys perceive parental psychological control as distressing, while girls tend to perceive the high level of anxious rearing from their mothers as distressing, both of which are linked with higher rates of body complaints. In contrast, their father’s anxious monitoring is experienced as positive by daughters, resulting in fewer body symptoms. Fathers’ lower scores in anxious rearing may have been perceived as appropriate and protective. These findings highlight the different roles that fathers and mothers play during adolescence [44]. However, it is important to consider the cross-sectional nature of our data, as the relationship between parenting style and body complaints may be bi-directional. Such bi-directional influences have been observed in various countries [10].

Overall, the associations between perceived parental behavior and body complaints were relatively weaker compared to the influence of cultural factors. In fact, cultural values and dimensions are adopted and implemented in families, and thus have a direct influence on young people. This may explain why the influence of culture was even stronger than that of family. We suspect that in families from cultures that do not prioritize adolescent independence, the open expression of distress may be hindered, leading to the expression of distress via body complaints. This highlights the role of the macrosystem level in Bronfenbrenner’s model [9], such as societal belief systems, cultural norms, ideologies, policies, or laws that indirectly shape healthy adolescent development. Across the different countries, the level of reported body complaints varied as expected. How these variations come about was partly explained in this study by taking cultural variation and variations in parenting style into account. Understanding the precise mechanisms by which each cultural indicator interacts with family variables to influence the development of somatic complaints among adolescents presents an important avenue for future research.

Body symptoms are seldom linked with organic disorders, but rather an expression of the difficulty to discern and regulate one’s own emotions [6,45]. These symptoms are crucial clinical warning signs, which, if not tended to, may persist into adulthood, herald subsequent mental illness like somatization, and may result in frequent consultation of medical services [1,3,46]. Our findings therefore carry important implications for prevention and intervention in adolescent health while being mindful of gender and cultural differences. This is important because we have increasing cultural diversity in Western cultures, and health professionals should be sensitive to cultural variations in the expression of stress, for example, in the form of body complaints. This affects both prevention and possible therapeutic approaches because there are obviously other causes than physical ones. It is further important to note that, regardless of cultural variations, we have very high rates of body complaints among girls, making them a particularly vulnerable group.

## 5. Conclusions, Limitations, and Future Directions

This study presents several strengths, including the investigation of a large sample of same-aged adolescents from eight different countries who shared a comparable developmental context. All were living in large university cities, and all were attending school. Future studies should include adolescents with greater socioeconomic variation and living circumstances within countries. Using Hofstede’s criteria as indicators of cultural differences among the different countries served as a first rough indicator of the influence of culture on mental health. It is important to acknowledge that the validity of some of the cultural dimensions has been questioned [47,48]. Additionally, in future studies, the validity of the body complaints scale should be tested in more detail, and measurement variance should be established. Moreover, given the culture-specific nature of somatization, more refined methods should be considered, as questionnaire data may not adequately capture culture-specific somatic symptoms [49]. In future studies, illness narratives could be collected from diverse ethnocultural groups to broaden our understanding of body complaints in different cultures. Furthermore, it would be of merit to assess the multiple meanings of body symptoms and the diverse psychological and social functions they serve. Including the perspective of parents on their parenting behavior would also contribute valuable insights. Finally, it is important to note that due to the cross-sectional design of our study, causality cannot be inferred. A longitudinal study design would allow for clearer predictions of whether somatic complaints are indeed influenced by certain parenting methods or family constellations. Regarding the statistical analyses, we decided against directly incorporating gender as a predictor into a single regression model but analyzed separate gender models. This way, we were able to obtain more fine-grained insights of gender differences beyond the association of gender and somatic complaints. 

The findings of this study demonstrate that the associations between parental behaviors and adolescents’ physical health were found in diverse international contexts. The expression of distress via body complaints is associated largely with cultural factors, whereas the association with family factors varied by gender. Notably, the negative effects of dysfunctional parental styles, such as psychological control and anxious monitoring, on body complaints were consistent across cultures. Parental intrusive behavior (for boys) and intrusive and monitoring behavior by mothers (for girls) was perceived as distressing and linked with higher rates of body complaints.

## Figures and Tables

**Table 1:** Sample characteristics separately for countries.

	Argentina n = 570	France n = 177	Germany n = 153	Greece n = 409	Pakistan n = 160	Peru n = 285	Poland n = 294	Turkey n = 379
	*M (SD)*	*M (SD)*	*M (SD)*	*M (SD)*	*M (SD)*	*M (SD)*	*M (SD)*	*M (SD)*
**Somatic complaints (boys)**	3.48 (0.21)	4.17 (0.33)	7.59 (0.38)	1.09 (0.23)	6.84 (0.35)	5.18 (0.28)	3.60 (0.30)	2.18 (0.22)
**Somatic complaints (girls)**	4.31 (3.53)	7.16 (3.75)	8.01 (2.70)	2.32 (3.14)	8.10 (4.11)	8.43 (4.56)	6.16 (3.49)	2.59 (3.28)
**Age**	15.48 (0.50)	15.51 (0.37)	14.88 (0.79)	15.18 (0.40)	15.58 (0.60)	15.60 (0.51)	15.61 (0.50)	14.86 (0.73)
**No of siblings**	2.35 (1.60)	1.59 (1.14)	1.11 (0.98)	1.37 (0.97)	3.39 (1.71)	1.51 (1.03)	1.35 (1.17)	1.23 (1.01)
	%	%	%	%	%	%	%	%
**Gender (female)**	57.4	51.7	57.7	56.7	50.0	58.2	64.1	51.2
**Family status**								
Two-parent	73.7	66.5	82.7	87.3	87.8	77.6	81.8	92.2
Single-parent	17.0	25.5	17.3	10.1	11.2	16.3	14.1	7.0
Stepparent	7.0	7.0	0.0	2.6	0.7	5.8	3.7	0.8
Other	2.6	1.2	0.0	0.0	0.3	0.3	0.3	0.0
**Self-rated SES**								
High	2.5	20.1	25.5	38.1	35.4	11.7	42.4	36.5
Average	88.7	78.7	49.7	59.4	64.6	77.0	56.5	62.4
Low	8.8	1.1	24.8	2.5	0.0	11.3	1.1	1.1
**Mother education**								
None	1.4	1.7	3.9	0	25.8	1.1	1.1	0
Primary/secondary	57.4	13.3	51.0	2.7	13.9	0.7	16.4	4.5
High school	41.2	37.6	43.1	55.2	21.2	24.7	35.8	22.8
Undergraduate/graduate	-	47.7	-	42.1	39.1	73.5	46.6	72.7
**Father education**								
None	2.2	1.7	3.3	0	3.2	0.7	0.4	0.3
Primary/secondary	65.8	23.7	54.2	6.2	11.5	0.7	27.1	1.6
High school	32.0	34.7	41.2	59.8	22.9	22.0	34.4	14.6
Undergraduate/graduate	-	39.9	-	34.1	62.4	76.5	38.2	83.6

**Table 2:** Hierarchical Regression Analysis on Predicting Somatic Complaints—Boys.

Steps/Blocks and Variables	Step 1 β	Step 2 β	Step 3 β	Step 4 β	Model Change		
					Adjusted R^2^	F	*p*
1st step/block							
Age	0.04	0.03	0.04	−0.01			
Two-parent vs. single-parent	0.09 **	0.10 **	0.10 **	0.09 **			
Two-parent vs. stepparent	−0.02	−0.01	<−0.01	−0.01			
Mother employment status	0.04	0.04	0.04	0.06 *			
Father employment status	−0.04	−0.04	−0.02	−0.03			
Standard of living	−0.09 **	−0.09 **	−0.09 **	−0.02			
Number of siblings	0.08 *	0.08 *	0.06	−0.03	0.03	10.472	<0.001
2nd step/block							
Mother support		0.05	0.07	0.04			
Mother psych. control		0.22 ***	0.09 *	0.10 *			
Mother anxious rearing		−0.04	−0.06	−0.04	0.04	17.401	<0.001
3rd step/block							
Father support			−0.03	−0.08 *			
Father psych. control			0.24 ***	0.11 **			
Father anxious rearing			0.01	0.07	0.04	9.397	<0.001
4th step/block							
Power distance				−0.08			
Individualism				−0.20 ***			
Masculinity				0.27 ***			
Uncertainty avoidance				−0.48 ***	0.14	41.980	<0.001

Note: * *p* ≤ 0.05; ** *p* ≤ 0.01; *** *p* ≤ 0.001; parenting variables were centered at their means.

**Table 3:** Hierarchical Regression Analysis on Predicting Somatic Complaints—Girls.

Steps/Blocks and Variables	Step 1 β	Step 2 β	Step 3 β	Step 4 β	Model Change		
					Adjusted R^2^	F	*p*
1st step/block							
Age	0.17 ***	0.18 ***	0.19 ***	0.14 **			
Two-parent vs. single-parent	0.05	0.05	0.06 *	0.05			
Two-parent vs. stepparent	0.09 **	0.08 **	0.09 **	0.07 **			
Mother employment status	−0.02	−0.01	−0.01	0.01			
Father employment status	0.01	0.02	0.03	0.01			
Standard of living	−0.11 ***	−0.11 ***	−0.11 ***	−0.08 **			
Number of siblings	0.05	−0.03	0.04	−0.05	0.06	10.472	<0.001
2nd step/block							
Mother support		−0.05	−0.07 *	−0.08 *			
Mother psych. control		0.11 **	0.03	−0.01			
Mother anxious rearing		0.10 **	0.14 ***	0.14 ***	0.04	17.401	<0.001
3rd step/block							
Father support			0.04	−0.02			
Father psych. control			0.17 ***	0.13 ***			
Father anxious rearing			−0.12 ***	−0.10 **	0.02	9.397	<0.001
4th step/block							
Power distance				0.11 **			
Individualism				−0.28 ***			
Masculinity				0.30 ***			
Uncertainty avoidance				−0.47 ***	0.10	38.883	<0.001

Note: * *p* ≤ 0.05; ** *p* ≤ 0.01; *** *p* ≤ 0.001; parenting variables were centered at their means.

## Data Availability

The datasets generated and analyzed during the current study are not publicly available due to confidentiality reasons. Requests to access the datasets should be addressed to ISK.
